# Personal

**Published:** 1905-10

**Authors:** 


### PERSONAL.

Rear-Admiral S. N. Suzuki, surgeon-general of the Imperial Japanese Navy, is paying a visit to America, where he will sojourn the remainder of the year, during which time he will visit the principal cities. He is now in Washington and was entertained by the President at luncheon on Friday, the 6th instant. Surgeon-General Suzuki made his first appearance in public after his arrival in this country at the annual dinner of the American Association of Obstetricians and Gynecologists September 20, at the Hotel Astor, where he made an interesting speech, giving a brief account of the methods adopted to conserve the health and lives of the Japanese sailors during the late war. An extended account of this occasion will appear in a later issue of the JOURNAL.

---

DR. FRANK WHITEHILL HINKEL, of Buffalo, who paid a visit to Europe last Summer has returned and resumed his larngolozical practice.

---

DR. FLOYD S. CREGO, of Buffalo, who recently spent three-fourths of a year in Europe, has returned and resumed his professional practice, at 469 Delaware avenue, corner of Virginia street. Hours: 12 to 3. Sundays and evenings by appointment.

---

DR. ALBERT H. BRIGGS, lieutenant-colonel and surgeon 65th Regiment N. G. N. Y., was elected president of the Association of Military Surgeons of the United States at the annual meeting held at Detroit, September 26-29, 1905. Under the leadership of Lieutenant-Colonel Briggs this famous body of medical men will hold its next annual meeting at Buffalo.

---

DR. SYDNEY A. DUNHAM, of Buffalo, who has been in ill health for some time has recovered and resumed his professional practice, which is limited to office and sanitarium work. Parkside Sanitarium for selected nervous cases, 1392 Amherst street; down-town office 239 Delaware avenue. Hours, 3-4 p. m.

